# *Xanthosoma sagittifolium* is resistant to *Meloidogyne* spp. and controls *Meloidogyne enterolobii* by soil biofumigation

**DOI:** 10.21307/jofnem-2020-107

**Published:** 2020-10-26

**Authors:** Vanessa Alves Gomes, Fabíola de Jesus Silva, Eunice Maria Baquião, Luana Viana Faria, Júlio César Antunes Ferreira, Marcio Pozzobon Pedroso, Fernando Broetto, Silvia Renata Siciliano Wilcken

**Affiliations:** 1 Department of Plant Protection, São Paulo State University (UNESP/FCA), 18610-034, Botucatu-SP, Brazil; 2 Department of Plant Pathology, Federal University of Lavras (UFLA), 37200-000, Lavras-MG, Brazil; 3 Department of Chemistry, Federal University of Lavras (UFLA), CP3037, 37200-000, Lavras-MG, Brazil; 4 Department of Chemistry and Biochemistry, São Paulo State University – Biosciences Institute (UNESP/IBB), 18618-687, Botucatu-SP, Brazil

**Keywords:** Alternative control, Araceae, Host–parasitic relationship, Resistance, Root-knot nematode, Volatiles

## Abstract

*Meloidogyne* is a relevant plant-parasitic nematode that causes enormous damage. It is very challenging to control, and there are not many chemicals available on the market for that. As an alternative method of nematode control, biofumigation is increasingly gaining space. This research aimed to study the reaction of *Xanthosoma sagittifolium* to *Meloidogyne enterolobii*, *M. incognita*, and *M. javanica* and soil biofumigation with *X. sagittifolium* leaves for *M. enterolobii* control. The reaction test was performed in the populations 0 (control), 333, 999, 3,000, 9,000, 27,000 eggs and eventual juveniles. *X. sagittifolium* did not host the *Meloidogyne* species studied, even in a high population. *X. sagittifolium* leaves incorporated in soil at concentrations 0 (control), 0.45, 0.9, 1.8, 3.6 g were also studied to control *M. enterolobii*, and they were able to reduce galls and eggs. The number of galls and egg masses was reduced to a concentration of 1.8 g. In the maximum concentration, the number of galls was less than 15 galls, and the eggs were also reduced to less than 200 eggs. As these macerates emitted nematicidal volatile organic compounds (VOCs) against *M. enterolobii*, it reduced the infectivity and reproduction of nematodes.

Plant-parasitic nematodes cause significant damage in tropical and subtropical agriculture. Among them, the *Meloidogyne* species, also known as root-knot nematode, affect agricultural production (Jones et al., 2011). The biggest problem in areas infested with these nematodes is the difficulty of managing and controlling them. Many nematicidal products have been withdrawn from the market due to their high toxicity to human beings and the environment (Sousa et al., 2015). Consequently, there is an increasing demand for lower-cost, high-efficiency, and sustainable new control methods (Subbotin and Chitambar, 2018).

Antagonistic plants have proved to be efficient in controlling plant-parasitic nematodes when used in crop rotation, extracts or pies in direct application to the soil, or soil biofumigation (Caboni et al., 2013; Babaali et al., 2017; Xie et al., 2017; Chetia et al., 2019). Biofumigation is an effective method to control soil pathogens, such as nematodes. This technique consists of macerating the plant and putting this macerate in contact with the infested soil. When biofumigation is performed, it is possible to potentiate the effect of compounds in the plants. Macerate of broccoli, cauliflower, papaya, crambe, castor bean, neem, and mustard showed nematicidal potentials (Ploeg and Stapleton, 2001; Barros et al., 2014; Tavares-Silva et al., 2017; Pedroso et al., 2019a; Gomes et al., 2020).

Natural compounds in some plant species may control nematodes. Several plant compounds have antimicrobial, insecticidal, or nematicidal action (Isman, 1999). These compounds include volatile organic compounds (VOCs), which consist of molecules with up to 20 carbon atoms with a high vapor pressure that cross membranes freely and release the vapors into the atmosphere or soil without a diffusion barrier (Dudavera et al., 2006). Plants produce several compounds known as secondary metabolites (Pichersky and Gang, 2000), with 1% being VOCs such as terpenoids, phenylpropanoids, benzenoids, fatty acid derivatives, and amino acid derivatives (Dudavera and Pichersky, 2008). Brassicas produce isothiocyanates by glucosinolate degradation (Price et al., 2005); garlic has allicin emission (Slusarenko et al., 2008) and neem, peas, mustard, velvet bean, and jack bean emit toxic VOCs to *Meloidogyne* (Barros et al., 2014). One advantage of using plants in nematode control is that they have fewer toxic substances than chemical control. Using plants to control nematodes instead of synthetic chemicals can be an effective and sustainable option. Usually, plants have no harmful effects on non-target organisms, and they are not persistent in the environment. Therefore, they are indicated for phytopathogens control (Jardim et al., 2018; Pedroso et al., 2019a; Gomes et al., 2020; Silva et al., 2020; Silva et al., 2020b).

The Araceae family has a wide variety of species, including the *Xanthosoma sagittifolium*. There is much confusion within this genus, with the same plant being given more than one species common name depending on the location where the plant is. These plants are native to South America, which grow mainly in regions of tropical and subtropical climate. Their cultivation is widespread, and they are intensively grown and consumed in Central American, Africa, and Asia (Seganfredo et al., 2001). Many species in this family are highly toxic, such as *Xanthosoma sagittifolium, Epipremnum pinnatum, Colocasia esculenta, Monstera deliciosa Colocasia antiquorum*, and *Dieffenbachia picta,* due to the presence of calcium oxalate (Sena et al., 2017). This substance participates in the toxic mechanism, causing injury and exposing the organism of the individual to the toxic substance (Ilarslan et al., 1997; de Oliveira and Pasin, 2017). The *X. sagittifolium* is composed of proteins, carbohydrates, vitamins, thiamine, riboflavin, niacin, fibers, among others. However, it can also present antinutritional factors, substances such as oxalates, proteinase inhibitors, phytates, tannins, alkaloids, steroids, and cyanogenic glycosides (Puiatti, 2002; Lewu et al., 2010). A study with rhizome extract from *X. sagittifolium* was efficient in reducing hatching and causing mortality of *M. megadora* (Galhano et al., 1997).

*M. enterolobii* is a relevant nematode because it causes damage to several hosts, most of them resistant to other nematode species (Bitencourt and Silva, 2010). This species has higher virulence and reproductive potential than other *Meloidogyne* species (Brito et al., 2015). *M. enterolobii* has been reported in many countries such as Mexico, in watermelon plants (Ramírez-Suárez et al., 2014), in jackfruit, tomato, and pepper in Florida, in the USA (Brito et al., 2015) and yam in Nigeria, with irreversible damage to these crops (Kolombia et al., 2016). In Brazil, this nematode has been found in several states, parasitizing guava (Silva et al., 2008), and in vegetables, mainly tomatoes and peppers (Carneiro et al., 2006). The nematode can break the resistance conferred by Mi-1 gene to *M. arenaria*, *M. incognita*, and *M. javanica* in tomato (Fargette et al., 1994), besides soybean ‘Forrest’ and sweet potato ‘CDH’ (Carneiro et al., 2001; Cantu et al., 2009; Bitencourt and Silva, 2010; Melo et al., 2011), and ‘Prata’ bell pepper (Carneiro et al., 2006).

Considering the great potential of crop rotation and soil biofumigation for plant-parasitic nematodes control, this study aimed to verify the reaction of *X. sagittifolium* to root-knot nematode species, *Meloidogyne incognita*, *Meloidogyne javanica*, and *M. enterolobii*; and the effectiveness of soil biofumigation with *X. sagittifolium* in controlling *M. enterolobii*. Furthermore, a gas chromatography analysis coupled to mass spectrometry was performed to identify the possible VOCs emitted by *X. sagittifolium* leaves, which can be involved in nematode control.

## Materials and methods

The experiments were carried out at the Laboratories of Nematology and greenhouses of São Paulo State University – UNESP/FCA – Botucatu, SP, Brazil. The nematode populations used as inoculums were obtained from pure populations of *Meloidogyne enterolobii, M. incognita*, and *M. javanica* maintained in tomato plants cv Santa Clara in greenhouse. To extract the eggs, infected roots were washed in tap water, chopped into 1 to 2 cm pieces, and grounded in a blender with 0.5% NaClO solution for approximately 20 sec. Finally, the material was placed into a 60-mesh screen over a 500-mesh screen, where the eggs were collected in water (Hussey and Barker, 1973 modified by Boneti and Ferraz, 1981).

*X. sagittifolium* rhizomes, obtained from Jardim de Minas Company, were partly used in experiments of *X. sagittifolium* suitability to *M. enterolobii, M. incognita*, or *M. javanica,* and the remaining was cultivated in a greenhouse to obtain leaves used in biofumigation experiments with *M. enterolobii* to represent this genus. Hence, the leaves were taken from the plants, sterilized using water solution with 2.0% bleach for 1 min, and double washed in distilled sterile water. After removing the excess water with a paper towel, they were macerated in a blender to be used immediately in the experiments.

### X. sagittifolium reaction to Meloidogyne spp.

The experiment was carried out in spring–summer in a greenhouse under controlled temperature conditions so as not to exceed 30°C, and in this period, there was no occurrence of low temperatures. The plants were subjected to daily irrigation and fertilization, according to the crop needs. Regarding diseases and pests, no problems were observed during the experiments, as the greenhouse was disinfected, and offered suitable conditions for the crop. The rhizomes were soaked in water for 12 hr and then transplanted to pots containing 2 L of autoclaved substrate composed of a mixture of soil, sand and organic matter (1:2:1). *Meloidogyne* spp. eggs suspension was prepared as described above. After one month, when the thaw had already developed, the researchers drilled holes in the soil with a glass stick around the plants’ roots, and inoculated the eggs’ suspension with a pipette for each treatment. The treatments were: 0 (control), 333, 999, 3,000, 9,000, 27,000 eggs and eventual juveniles and five replications, each plot consisting of one plant. ‘Rutgers’ tomato plants were used to attest the viability of the *M. enterolobii, M. incognita*, or *M. javanica* inoculum (1,000 eggs and eventual juveniles). The experiments consisted of a completely randomized experimental design with six different treatments and five repetitions. The experiments were evaluated 60 days after nematode inoculation. The galls and egg masses were evaluated according to the grade scale proposed by Taylor and Sasser (1978), where grade 0 = 0, 1 = 1 to 2, 2 = 3 to 10, 3 = 11 to 30, 4 = 31 to 100, and 5 = more than 100 galls or egg masses. The root systems were washed and processed separately, where the roots were chopped, and the eggs were extracted by Hussey and Barker’s (1973) technique, modified by Boneti and Ferraz (1981). The collected eggs were counted under an inverted light microscope (Leica, model DME). Finally, the reproduction factors of the nematodes were calculated (RF = final population/initial population). Plants with FR < 1 are considered resistant, and FR ≥ 1 are considered susceptible (Oostenbrink, 1966). The experiments were conducted separately and followed the same methodology described above for each nematode species, *M. enterolobii*, *M. incognita*, and *M. javanica*. The biofumigation test was performed only with the species *M. enterolobii* to represent the root-knot nematodes.

### Biofumigation with macerated X. sagittifolium leaves

*M. enterolobii* was used to represent root-knot nematodes species at biofumigation tests. The experiment was carried out in spring–summer in a greenhouse under controlled temperature conditions so as not to exceed 30°C, and in this period, there was no occurrence of low temperatures. The plants were subjected to daily irrigation and fertilization according to the crop needs. Regarding diseases and pests, no problems were observed during the experiments, as the greenhouse was disinfected and offered suitable conditions for the crop. The experiment was conducted with five treatments and five repetitions. Macerated *X. sagittifolium* leaves, obtained as described above, were incorporated into the commercial substrate (Carolina Soil – Pardinho, SP, Brazil) in amounts of about 0 (control), 0.45, 0.9, 1.8, 3.6 g corresponding the percentage of 0, 1, 2, 4, 8% and put at plastic cups (45 g). In total, 1,000 eggs of *M. enterolobii* in 2.0 ml of water was placed in the plastic cups together with the substrate and the macerated *X. sagittifolium* leaves and shaken with a glass stick until homogenization. The moisture of mixture in the plastic cups was adjusted to 60% of the field capacity (20 ml). The surface of the mixture was completely covered with plastic caps, thereby forming a gas chamber between the plastic caps and the substrate by emissions of VOCs (volatile organic compounds) from *X. sagittifolium* leaves macerates mixed to the substrate. The eggs were left exposed to the VOCs released from the mixture for three days. After that, the plastic caps were removed, and this mixture was placed in a styrofoam tray (76 cells, 120 cm^3^ each cell) with a 20-day-old tomato seedling cv. Santa Clara. Each cell in the styrofoam tray represents an experimental unity, with five treatments and five repetitions too. After 60 days, each plant was removed. The substrate was carefully removed from the roots by water, and the root system was placed on a paper towel to remove excess water, weighed, and the number of galls per gram of root was counted. Then, the roots were chopped, and the eggs were extracted by Hussey and Barker’s (1973) technique, modified by Boneti and Ferraz (1981). The collected eggs were counted in Peters slide under an inverted light microscope. Finally, the reproduction factor of the nematodes was calculated (RF = final population/initial population).

### Gas chromatography analysis of VOCs emitted by Xanthosoma sagittifolium leaves

Volatile identification was conducted at the Center of Analysis and Chemical Prospecting (CAPQ – Department of Chemistry/UFLA). Gas chromatography (GC-MS) was carried out, first, with the macerate of leaves of *X. sagittifolium*, and second, with the water exposed to the volatiles emitted by the macerate of the leaves of *X. sagittifolium* to verify whether the volatile organic compounds (VOCs) can be retained in water. Six replicates of 1.5 g of macerated *X. sagittifolium* leaves and six replicates of water in contact with *X. sagittifolium* leaves were prepared for analysis of VOCs. Samples were placed in 20 ml SPME Supelco vial, closed and kept in an incubation chamber at 28°C for three days. Three vials containing only sterile sand and three empty tubes were also analyzed as controls. Three days later, the volatiles from macerated *X. sagittifolium* leaves were extracted by SPME technique in headspace mode, using DVB/CAR/PDMS fiber (Divinylbenzene, Carboxen, Polydimethylsiloxane). The temperature and extraction time were 55°C to 250 rpm for 35 min, respectively. The GC-MS QP 2010 Ultra mass spectrometer (Shimadzu, Japan), equipped with AOC-5000 automatic liquid and gas injector (Shimadzu, Japan) and HP-5 column, was used for the separation and identification of VOCs (5% phenyl-95% dimethylsiloxane) of dimensions 30 m × 0.25 mm × 0.25 μm. The temperature of the injector was 250°C, the interface was 240°C, and the detector ion source was 200°C. The injector was operated in splitless mode or split 1: 2 mode, according to the peak intensity in the samples. As carrier gas He 5.0 was used as 1.0 ml.min^−1^. The temperature setting of the GC oven was 40 to 130°C at 3°C min^-1^ and then up to 240°C at 10°C min^-1^. To identify the VOCs in the samples, the mass spectrum of each chromatogram peak was extracted through the Automated Mass Spectral Deconvolution and Identification System (AMDIS) v. 2.63. The VOC identification was done by comparing the mass spectra of the sample peaks and the NIST library spectra by the Mass Spectral Search Program v. (NIST, Washington, DC, USA), and by comparing the experimentally obtained retention indices (RI Exp.) and the retention indices of the literature (RI Lit.) (Adams, 2007; Nist, 2013). The experimental retention indices were obtained by injecting a homologous series of alkanes. The comparison among the mass spectra was made only for peaks in which the similarity was greater than 80%.

### Statistical analysis

All experiments were repeated twice. The trials were arranged in a completely randomized design with five replicates per treatment. The data were previously submitted to normality tests (Shapiro–Wilk test) and homogeneity of variance of the errors (Bartlett’s test). Once the assumptions were met, the *F*-test was applied through analysis of variance (ANOVA). The experiments were analyzed separately. When the significance level (*P* < 0.05) occurred in biofumigation experiments, regression analysis was performed with the aid of SigmaPlot software (SigmaPlot 12.0, Systat Software Inc.) where the graphs were generated too. In *X. sagittifolium* reaction to *M. enterolobii, M. incognita*, and *M. javanica*, the variables were transformed by √*x* + 1 and when the significance level (*P* < 0.05) occurred, made Scott–Knott test was performed with the aid of Sisvar software (Ferreira, 2014).

## Results and discussion

We studied whether *X. sagittifolium* can be host the root-knot nematode. Due to its resistance characteristics, we also studied if *X. sagittifolium* can control *M. enterolobii*. Biofumigation with leaves of *X. sagittifolium* effectively suppressed *M. enterolobii* in tomato roots. Many species in Araceae family have highly toxic in their composition, and there are no studies about these plants. Based on the toxic composition of *X. sagittifolium* in these experiments and the study by Galhano et al. (1997), the authors believe in the potential of using this practice in the field, but further studies are needed to verify whether these plants could be used in agriculture to crop rotation and soil biofumigation.

In reaction tests, the researchers observed that there was no formation of galls and external egg masses of the nematodes. *X. sagittifolium* was unfavorable for *M. enterolobii, M. incognita*, and *M. javanica* multiplication, with reproductive factors less than one, or even equal to zero, being considered resistant to these species of root-knot nematodes (Tables 1-3). The plant continued to show resistance patterns even with the inoculation of a high nematode population. Plants of last treatment had few root systems in the presence of the nematode and these changes may occur as a plant survival strategy (Tables 1-3). The viability of the inoculum is proven in the tomato treatment, where the reproduction factor was 13.9, 12.35, and 10.6 for *M. enterolobii, M. incognita*, and *M. javanica*, respectively, being the only treatment that behaved as susceptible (Tables 1-3).

**Table 1.** Reaction test on *Xanthosoma sagittifolium* with *Meloidogyne enterolobii* under the six treatments and the tomato (indicator plant) showing susceptibility or resistance in the treatments based on the reproduction factor (RF).

		*M. enterolobii*				
Plant	Inoculated eggs^a^	Galls	Egg mass	Eggs	RF^b^	Classification
*X. sagittifolium*	0	0b	0b	0b	0b	Resistant
	333	0b	0b	0b	0b	
	999	0b	0b	0b	0b	
	3,000	0b	0b	0b	0b	
	9,000	0b	0b	316b	0.03b	
	27,000	0b	0b	1,076b	0.04b	
Tomato	1,000	4.80a	4.80a	13,876a	13.9a	Susceptible
CV (%)	–	3.00	3.00	42.78	10.41	–

**Notes:** ^a^Number of inoculated eggs; ^b^FR = final population/initial population. Plants with FR < 1 are considered resistant and FR ≥ 1 susceptible (Oostenbrink, 1966).

**Table 2.** Reaction test on *Xanthosoma sagittifolium* with *Meloidogyne incognita* under the six treatments and the tomato (indicator plant) showing susceptibility or resistance in the treatments based on the reproduction factor (RF).

		*M. incognita*				
Plant	Inoculated eggs^a^	Galls	Egg mass	Eggs	RF^b^	Classification
*X. sagittifolium*	0	0b	0b	0b	0b	Resistant
	333	0b	0b	0b	0b	
	999	0b	0b	0b	0b	
	3,000	0b	0b	0b	0b	
	9,000	0b	0b	0b	0b	
	27,000	0b	0b	0b	0b	
Tomato	1,000	4.80a	4.80a	12,350a	12.35a	Susceptible
CV (%)	–	3.00	3.00	40.86	14.86	–

**Notes:** ^a^Number of inoculated eggs; ^b^RF = final population/initial population. Plants with FR < 1 are considered resistant and FR ≥ 1 susceptible (Oostenbrink, 1966).

**Table 3.** Reaction test on *Xanthosoma sagittifolium* with *Meloidogyne javanica* under the six treatments and the tomato (indicator plant) showing susceptibility or resistance in the treatments based on the reproduction factor (RF).

		*M. javanica*				
Plant	Inoculated eggs^a^	Galls	Egg mass	Eggs	RF^b^	Classification
*X. sagittifolium*	0	0b	0b	0b	0b	Resistant
	333	0b	0b	0b	0b	
	999	0b	0b	240b	0.24b	
	3,000	0b	0b	326b	0.11b	
	9,000	0b	0b	360b	0.04b	
	27,000	0b	0b	582b	0.02b	
Tomato	1,000	4.80a	4.80a	10,592a	10.6a	Susceptible
CV (%)	–	3.00	3.00	56.59	14.19	–

**Notes:** ^a^Number of inoculated eggs; ^b^RF = final population/initial population. Plants with FR < 1 are considered resistant and FR ≥ 1 susceptible (Oostenbrink, 1966).

**Figure 1:**
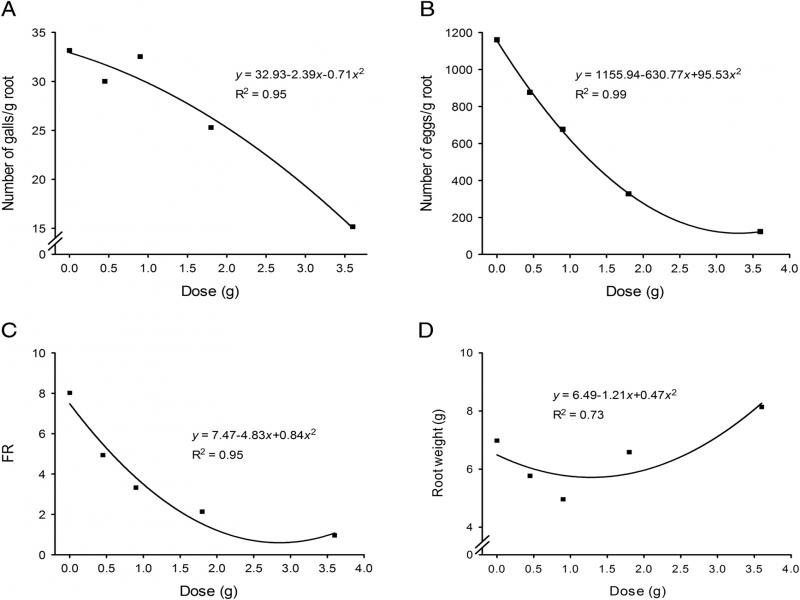
Biofumigation with *Xanthosoma sagittifolium* leaves macerates against *Meloidogyne enterolobii*. These experiments were done by incorporating *X. sagittifolium* leaves at different concentration (g/45 g) along with *M. enterolobii* eggs and planting tomato after three days of biofumigation. A: Number of galls. B: Number of eggs. C: Reproduction factor. D: Root weight. The experiments were done twice. Quadratic polynomial regression models, *p* < 0.01.

The biofumigation with *X. sagittifolium* had a significant effect on the number of *M. enterolobii* galls and eggs. So, *X. sagittifolium* has the potential to control *M. enterolobii*. By increasing the amount of the macerated *X. sagittifolium* leaves, the number of galls and eggs per gram of root reduced significantly (*p* < 0.05) (Fig. 1A, B). The reduction in the number of galls was more evident at the 1.8 g concentration, about 25 galls/g root. The reduction in the galls continued by increasing the *X. sagittifolium* macerate amount, reaching about 15 galls/g root with 3.6 g of macerate (Fig. 1A). Also, the reduction in the number of eggs became more expressive at the concentration of 0.9 g, about 600 eggs/g root. The reduction in the eggs number was observed at 0.45 g of macerate, reaching significant reductions, less than 200 eggs/g root in 3.6 g of macerate (Fig. 1B). Both results were compared to the values obtained in the control (0 g) (Fig. 1A, B).

When the reproduction factor was evaluated in biofumigation experiments, the value started to reduce in 0.45 g, reaching a reproduction factor lower or equal to one in the highest concentration (3.6 g). Significant reduction when compared to the control (0 g) with reproduction factor around eight (Fig. 1C). Regarding root weight, the analysis revealed that with increasing concentrations, there was an increase in root weight, when compared with de control (0 g) (Fig. 1D). Some studies have verified the efficiency of biofumigation in the control of nematodes with vegetable residues and even papaya seeds (Ploeg and Stapleton, 2001; Wang et al., 2009; Ntalli et al., 2011; Gomes et al., 2020). The reduction in gall and egg formation by applying 1.8 g.45 mL^−1^ of macerate represents about 60-ton ha^−1^ (1 ha × 15 cm depth = 1,500 m^3^). Generally, to biofumigate the soil with plants, the incorporation of 50-ton ha^−1^ is recommended (Ploeg and Stapleton, 2001). Although the *X. sagittifolium* application was higher than the general recommendation, these plants produce a sizeable aerial part that is still a good option for biofumigation.

As concentrations of *X. sagittifolium* leaves macerate increased, there was an increase in root weight. This happens because, with the reduction of the number of galls, the roots develop more and increase their root weight. Some plant VOCs have the potential to interfere with infectivity and reproduction of root-knot nematode, reducing theirs galls and eggs (Kihika et al., 2017; Jardim et al., 2018; Gomes et al., 2020; Silva et al., 2020a; Silva et al., 2020b) and in this work, the same effect is observed. Brassicas can be used in biofumigation as a feasible practice to reduce plant-parasite nematodes in crop fields (Ploeg, 2008; Lord et al., 2011; Chetia et al., 2019). However, further studies are needed to verify the best methodology in the field and the way that brassica works in the field in this cultural control practice. *X*. *sagittifolium* leaves studied in this work showed toxic activity against *M. enterolobii* that was comparable to the activity shown from broccoli. Thus, additional trials should be conducted using the *X. sagittifolium* to confirm whether their use as biofumigants is viable, because it is not a host to *M. enterolobii* (FR < 1), as we can see in the study of the host suitability of *X. sagittifolium* (Tables 1-3).

The GC-MS of the VOCs emitted by macerated *X. sagittifolium* leaves revealed 19 compounds in total. The compounds identified were categorized as low (‘+’), and medium to high (‘++’), by the intensity of the peaks detected in the chromatograms. The compounds identified with medium to high intensities were ethanol, acetone, 3-methyl-butanol, and 3-hexen-1-ol. The compounds propanol, ethyl acetate, 2-methyl-propanol, 3-penten-1-ol, 3-pentanone, acetoin, 2-methyl-butanol, 2,3-butanediol, ethyl 2-methyl-butanoate, hexanol, 3-methyl-butyl acetate, 3-octanone, 2-ethyl-hexanol, and dodecane occurred in small intensities in macerates of *X. sagittifolium* leaves, as well as a not identified compound (Table 4).

**Table 4.** Volatilome of *Xanthosoma sagittifolium* leaves by gas chromatography.

Compounds	RI exp^a^	RI Lit^b^	Intensity^c^ Macerated	Intensity^c^ Water
Ethanol	–	–	XX	XX
Acetone	–	–	XX	XX
Propanol	–	–	X	X
Ethyl acetate	609	628	X	X
2-methyl-propanol	615	622	–	X
3-penten-1-ol	687	686	X	X
3-pentanone	700	696	X	X
Acetoin	720	718	X	–
3-methyl-butanol	737	734	XX	XX
2-methyl-butanol	740	738	X	X
2,3-butanediol	811	811	X	–
Ethyl 2-methyl-butanoate	849	846	X	–
3-hexen-1-ol	856	857	XX	XX
Hexanol	871	867	X	X
3-methyl-butyl acetate	875	880	X	–
3-octanone	985	984	X	–
2-ethyl-hexanol	1,033	1,029	X	X
Not identified	1,189	–	X	–
Dodecane	1,199	1,200	X	–

**Notes:** ^a^Calculated retention indices by injecting a homologous series of alkanes; ^b^Theoretical retention index according to the literature (ADAMS, 2007); ^c^X: low intensity; XX: medium to high intensity.

The chromatographic analysis revealed several compounds of the alcohol group: ethanol, propanol, 3-penten-1-ol, 3-methyl-butanol, 2-methyl-butanol, 2,3-butanediol, 3-hexene-1-ol, hexanol, and 2-ethyl-hexanol. It is known that ethanol could result from the fermentation of plants (Olsson and Hahn-Hägerdal, 1996). However, the ethanol (EtOH), when tested by applying the isolate compound, is known to have a nematicidal potential (Silva et al., 2017; Pedroso et al., 2019b). Thus, regardless of the origin of the compound, it behaves as toxic to nematodes. Concentrations of EtOH below 5% by volume had a toxic effect on the *Caenorhabditis elegans,* and this nematode is the model nematode for biology studies and development of the nematode (Patananan et al., 2015). Other studies have shown that aqueous EtOH solutions (70% volume) and their vapors reduced hatching of J2 of *M. incognita*. There was also large reduction of galls and eggs in the root system when 40 ml of EtOH (40 and 70%) were applied in the soil with *M. incognita*. Water exposed to EtOH vapors for 1 hr became toxic and caused 100% J2 mortality in 12 hr (Silva et al., 2017). Another study reveals that ethanol was responsible for the control of *Heterodera glycines*. Soil infested by *H. glycines* with ethanol reduced infectivity by almost 100% and number of eggs by about 67% at ethanol concentrations of 48% and 72%, respectively. Ethanol concentration at 48% can reduce the lipid content of J2. So, the ethanol is toxic to *H. glycines* in low concentrations, and affects their pathogenic behavior, instead of merely reducing lipids (Pedroso et al., 2019b). Thus, these compounds may present toxicity to nematodes and act to control nematodes, not only of the genus *Meloidogyne*.

Acetone, 3-pentanone, and octanone are from the ketone class. Studies show that aliphatic ketones from *Ruta chalepensis* (Rutaceae) induce paralysis on root-knot nematodes (Ntalli et al., 2011). This suggests that this group has some phytonematoid toxicity. We also found dodecane and esters, such as ethyl acetate, ethyl 2-methyl-butanoate, 3-methyl-butyl acetate in the *X. sagittifolium* leaves. Little is known about the toxicities of these molecules and whether their contact with phytonematoids may cause any control. Further studies on these secondary compounds found in GC-MS are needed to determine and prove which indeed have *M. enterolobii* nematode toxicity and can be used to control this plant-parasite nematode.

**Figure S1:**
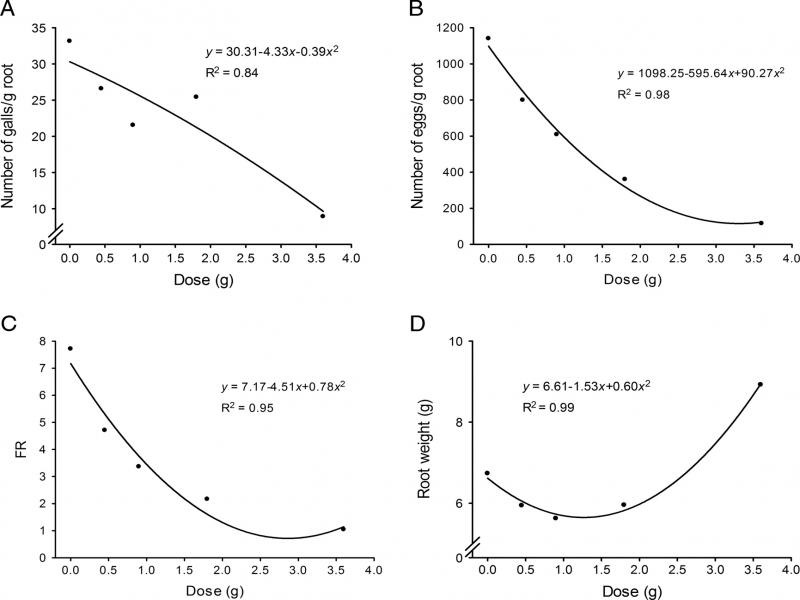
Biofumigation with *Xanthosoma sagittifolium* leaves macerates against *Meloidogyne enterolobii*. These experiments were done by incorporating *X. sagittifolium* leaves at different concentration (g/45 g) along with *M. enterolobii* eggs and planting tomato after three days of biofumigation. A: Number of galls. B: Number of eggs. C: Reproduction factor. D: Root weight. Experiment two. Quadratic polynomial regression models, *p* < 0.01.

In conclusion, this research demonstrated, for the first time, the performance of *X. sagittifolium* in the control of *M. enterolobii*. The life cycle of *M. enterolobii* was interrupted when had *X. sagittifolium* plants. *X. sagittifolium* is toxic to this nematode by reducing eggs and galls and this can be due the secondary compounds present in its composition, but tests with the secondary compounds are necessary to prove. Further studies of this interaction of the compounds with the soil microbiota are necessary to verify whether they are altered by it before acting on the nematode control. Even though *X. sagittifolium* is little known globally, this is an ordinary plant that adapts to many environments and proliferates rapidly. Therefore, the fact of *X. sagittifolium* leaves are toxic to *M. enterolobii* in low amounts could allow crop rotation with this plant. Then, after the crop rotation, it is possible to incorporated this plant into infested soils with *Meloidogyne* spp. Furthermore, the exploitation of VOCs from this plant could reveal novel toxic compounds able to be applied as nematicidal products. To prove it, these compounds should be tested separately in subsequent studies (Fig. S1).

## References

[ref001] Adams, R. P. 2007. Identification of essential oil components by gas chromatography/mass spectrometry Carol Stream: Allured Publishing Corporation.

[ref002] Babaali, D. , Roeb, J. , Hammache, M. and Hallmann, J. 2017. Nematicidal potential of aqueous and ethanol extracts gained from *Datura stramonium*, *D. innoxia* and *D. tatula* on *Meloidogyne incognita* . Journal of Plant Diseases and Protection 124:339–348.

[ref003] Barros, A. F. , Campos, V. P. , Silva, J. C. P. , Pedroso, M. P. , Medeiros, F. H. V. , Pozza, E. A. and Reale, A. L. 2014. Nematicide activity of volatile organic compounds emitted by *Brassica juncea, Azadirachta indica, Canavalia ensiformis, Mucuna pruriens* and *Cajanus cajan* against *Meloidogyne incognita* . Applied Soil Ecology 80:34–43.

[ref004] Bitencourt, N. V. and Silva, G. S. 2010. Reprodução de *Meloidogyne enterolobii* em Olerícolas. Nematologia Brasileira 34:181–183.

[ref005] Boneti, J. I. S. and Ferraz, S. 1981. Modificação do método de Hussey and Barker para extração de ovos de *Meloidogyne exigua* de cafeeiro. Fitopatologia Brasileira 6:553.

[ref006] Brito, A. , Smith, T. and Dickson, D. W. 2015. First report of *Meloidogyne enterolobii* infecting *Artocarpus heterophyllus* Worldwide. Plant Disease 99:1284.

[ref007] Caboni, P. , Saba, M. , Tocco, G. , Casu, L. , Murgia, A. , Maxia, A. , Menkissogolu-Spiroudi, U. and Ntalli, N. 2013. Nematicidal activity of mint aqueous extracts against the root-knot nematode *Meloidogyne incognita* . Journal of Agricultural and Food Chemistry 61:9784–9788. 24050256 10.1021/jf403684h

[ref008] Cantu, R. R. , Wilcken, S. R. S. , Rosa, J. M. O. and Goto, R. 2009. Reação de porta-enxertos de tomateiros a *Meloidogyne mayaguensis* . Suma Phytopathologica 35:216–218.

[ref009] Carneiro, R. M. D. G. , Moreira, W. A. , Almeida, M. R. A. and Gomes, A. C. M. M. 2001. Primeiro registro de *Meloidogyne mayaguensis* em goiabeira no Brasil. Nematologia Brasileira 25:223–232.

[ref010] Carneiro, R. M. D. G. , Almeida, M. R. A. , Braga, R. S. , Almeida, C. A. and Gloria, R. 2006. Primeiro registro de *Meloidogyne mayaguensis* parasitando plantas de tomate e pimentão resistentes à meloidoginose no estado de São Paulo. Nematologia Brasileira 30:81–86.

[ref011] Chetia, A. , Gogoi, P. , Roy, P. and Goswani, M. 2019. Antagonistic plants as a tool of nematode management. Journal of Medicinal Plants Studies 7:113–116.

[ref012] de Oliveira, R. R. and Pasin, L. A. A. P. 2017. Ocorrência de oxalato de cálcio em plantas não relatadas como tóxicas. Revista Científica da FEPI - Revista Científica Universitas 6:1–4.

[ref013] Dudavera, N. and Pichersky, E. 2008. Metabolic engineering of plant volatiles. Current Opinion in Biotechnology 19:181–189. 18394878 10.1016/j.copbio.2008.02.011

[ref014] Dudavera, N. , Negre, F. , Nagegowda, D. A. and Orlova, I. 2006. Plant volatiles: recent advances and future perspectives. Critical Reviews in Plant Science 25:417–440.

[ref015] Fargette, M. , Davies, K. G. , Robinson, M. P. and Trudgill, D. L. 1994. Characterisation of resistance breaking *Meloidogyne incognita*-like populations using lectins, monoclonal antibodies and spores of *Pasteuria penetrans* . Fundamental and Applied Nematology 17:537–542.

[ref016] Ferreira, D. F. 2014. Sisvar: a computer statistical analysis system. Ciência e Agrotecnologia 35:1039–1042.

[ref017] Galhano, C. I. C. , Ryan, M. F. , Santos, M. S. N. and Starisky, G. 1997. Interactions between Tannia (*Xanthosoma sagittifolium*) and the root-knot nematodes, *Meloidogyne megadora* and *M. javanica* . Nematropica 27:7–17.

[ref018] Gomes, V. A. , Campos, V. P. , Silva, J. C. P. , Silva, F. J. , Silva, M. F. and Pedroso, M. F. 2020. Activity of papaya seeds (*Carica papaya*) against *Meloidogyne incognita* as a soil biofumigant. Journal of Pest Science 93:783–792.

[ref019] Hussey, R. S. and Barker, K. R. A. 1973. Comparison of methods of collecting inocula of *Meloidogyne* spp., including a new technique. Plant Disease Reporter 57:1025–1028.

[ref020] Ilarslan, H. , Palmer, R. G. , Imsande, J. and Horner, H. T. 1997. Quantitative determination of calcium oxalate and oxalate in developing seeds of soybean (Leguminosae). American Journal of Botany 84:1042–1046. 21708659

[ref021] Isman, M. 1999. Pesticides based on plant essential oils. Pesticide Outlook 10:68–72.

[ref022] Jardim, I. N. , Oliveria, D. F. , Silva, G. H. , Campos, V. P. and Souza, P. E. 2018. (E)-cinnamaldehyde from the essential oil of *Cinnamomum cassia* controls *Meloidogyne incognita* in soybean plants. Journal of Pest Science 91:479–487.

[ref023] Jones, J. , Gheysen, G. and Fenoll, C. 2011. “Genomics and molecular genetics of plant-nematode interactions”, In Nicol, J. M. (Ed.), Current Nematode Threats to World Agriculture. Dordrecht: Springer Science, pp. 632–636.

[ref024] Kihika, R. , Murungi, L. K. , Coyne, D. , Hassanali, A. , Teal, P. E. and Torto, B. 2017. Parasitic nematode *Meloidogyne incognita* interactions with different *Capsicum annum* cultivars reveal the chemical constituents modulating root herbivory. Scientific Reports 7:1–10. 28588235 10.1038/s41598-017-02379-8 PMC5460232

[ref025] Kolombia, Y. A. , Kumar, P. L. , Claudius-Cole, A. O. , Karssen, G. , Viane, N. , Coyne, D. and Bert, W. 2016. First report of *Meloidogyne enterolobii* causing tuber galling damage on white yam (*Dioscorea rotundata*) in Nigeria. Plant Disease 100:2173.

[ref026] Lewu, M. N. , Yakubu, M. T. , Adebola, P. O. and Afolayan, A. J. 2010. Effect of accessions of *Colocasia esculenta*-based diets on the hepatic and renal functional indices of weanling wistar rats. Journal of Medicinal Food 13:1210–1215. 20828320 10.1089/jmf.2009.0211

[ref027] Lord, J. S. , Lazzeri, L. , Atkinson, H. J. and Urwin, P. E. 2011. Biofumigation for control of pale potato cyst nematodes: activity of brassica leaf extracts and green manures on *Globodera pallida* in vitro and in soil. Journal of Agricultural and Food Chemistry 59:7882–7890. 21718044 10.1021/jf200925k

[ref028] Melo, O. D. , Maluf, W. R. , Gonçalves, R. J. S. , Gonçalves Neto, A. C. , Gomes, L. A. A. and Carvalho, R. C. 2011. Triagem de genótipos de hortaliças para resistência a *Meloidogyne enterolobii* . Pesquisa Agropecuária Brasileira 46:829–835.

[ref029] Nist 2013. Wnt signaling. (September 20, 2019). Chemistry Webook-National Institute of Standards and Technology, ed Wormbook, available at: http://webbook.nist.gov/chemistry/.

[ref030] Ntalli, N. G. , Manconi, F. , Leonti, M. , Maxia, A. and Caboni, P. 2011. Aliphatic ketones from *Ruta chalepensis* (Rutaceae) induce paralysis on root-knot nematodes. Journal of Agricultural and Food Chemistry 59:7098–7103. 21631118 10.1021/jf2013474

[ref031] Olsson, L. and Hahn-Hägerdal, B. 1996. Fermentation of lignocellulosic hydrolysates for ethanol production. Enzyme and Microbial Technology 18:312–331.

[ref032] Oostenbrink, M. 1966. Major characteristics of the relation between nematodes and plants. Mendelingen Landbouwhogeschool Wageningen 66:1–46.

[ref033] Patananan, A. N. , Budenholzer, L. M. , Eskin, A. , Torres, E. R. and Clarke, S. G. 2015. Ethanol-induced differential gene expression and acetyl-CoA metabolism in a longevity model of the nematode *Caenorhabditis elegans* . Experimental Gerontology 61:20–30. 25449858 10.1016/j.exger.2014.11.010 PMC4289658

[ref034] Pedroso, L. A. , Campos, V. P. , Barros, A. F. , Justino, J. C. and Paula, L. L. 2019a. Activity against *Meloidogyne incognita* of volatile compounds produced during amendment of soil with castor bean cake. Journal of Nematology 1:1–10.

[ref035] Pedroso, L. A. , Campos, V. P. , Barros, A. F. , Silva, J. C. P. , Assis, G. M. and Ribeiro, C. R. 2019b. Nematicidal activity of ethanol solutions on soybean cyst nematode *Heterodera glycines* . Journal of Nematology 1:1–11. 10.21307/jofnem-2019-021 PMC6929653 31088033

[ref036] Pichersky, E. and Gang, D. R. 2000. Genetics and biochemistry of secondary metabolites in plants: an evolutionary perspective. Trends in Plant Science 5:439–445. 11044721 10.1016/s1360-1385(00)01741-6

[ref037] Ploeg, A. 2008. “Biofumigation to manage plant-parasitic nematodes”, In Ciancio, A. (Ed.), Integrated Management and Biocontrol of Plant Pests and Diseases, Vol. 2. Dordrecht: Springer Science, pp. 239–248.

[ref038] Ploeg, A. T. and Stapleton, J. J. 2001. Glasshouse studies on the effects of time, temperature and amendment of soil with broccoli plant residues on the infestation of melon plants by *Meloidogyne incognita* and *M. javanica* . Journal of Nematology 3:855–861.

[ref039] Price, A. J. , Charron, C. S. , Saxton, A. M. and Sams, C. E. 2005. Allyl isothiocyanate and carbon dioxide produced during degradation of *Brassica juncea* tissue in different soil conditions. HortScience 40:1734–1739.

[ref040] Puiatti, M. 2002. “Manejo da cultura do taro”, In Carmo, C. A. S. (Ed.), Inhame e taro: sistema de produção familiar. Vitória: Incaper, pp. 203–252.

[ref041] Ramírez-Suárez, A. , Rosas-Hernández, L. , Alcasio-Rangel, S. and Powers, T. 2014. First report of the root-knot nematode *Meloidogyne enterolobii* parasitizing watermelon from Veracruz, Mexico. Plant Disease 98:428. 10.1094/PDIS-06-13-0636-PDN 30708415

[ref042] Seganfredo, R. , Finger, F. L. , Barros, R. S. and Mosquim, P. R. 2001. Influência do momento de colheita sobre a deterioração pós-colheita em folhas de taioba. Horticultura Brasileira 19:184–187..

[ref043] Sena, S. B. , Rocha, C. L. D. , Santana, D. A. O. , Aguiar, L. R. and Souza, A. C. R. 2017. Plantas tóxicas: análise *in loco* da existência no bairro areal em Porto Velho-Ro. Saber Científico V:1–13.

[ref045] Silva, J. C. P. , Campos, V. P. , Freire, E. S. , Terra, W. C. and Lopez, L. E. 2017. Toxicity of ethanol solutions and vapours against *Meloidogyne incognita* . Journal of Nematology 19:271–280.

[ref044] Silva, J. C. T. , Oliveira, R. D. L. , Jham, G. N. and Aguiar, N. D. C. 2008. Effect of neem seed extracts on the development of the soybean cysts nematode. Tropical Plant Pathology 33:171–179.

[ref046] Silva, M. F. , Campos, V. P. , Barros, A. F. , Silva, J. C. P. , Pedroso, M. P. , Silva, F. J. , Gomes, V. A. and Justino, J. C. 2020a. Medicinal plant volatiles applied against the root-knot nematode *Meloidogyne incognita* . Crop Protection 130:105057.

[ref047] Silva, M. F. , Campos, V. P. , Barros, A. F. , Terra, W. C. , Pedroso, M. P. , Gomes, V. A. , Ribeiro, C. R. and Silva, F. J. 2020b. Volatile emissions of watercress (*Nasturtium officinale*) leaves and passion fruit (*Passiflora edulis*) seeds against *Meloidogyne incognita* . Pest Management Science 76:1413–1421. 31625270 10.1002/ps.5654

[ref048] Slusarenko, A. J. , Pastel, A. and Portz, D. 2008. Control of plant disease by natural products: allicin from garlic as a case study. European Journal of Plant Pathology 121:313–322.

[ref049] Sousa, R. M. O. F. , Rosa, J. S. , Silva, C. A. , Almeida, M. T. M. , Novo, M. T. , Cunha, A. C. and Fernandes-Fereira, M. 2015. Larvicidal, molluscicidal and nematicidal activities of essential oils and compounds from *Foeniculum vulgare* . Journal of Pest Science 88:413–426.

[ref050] Subbotin, A. S. and Chitambar, J. J. 2018. Plant parasitic nematodes in sustainable agriculture of North America. Northeastern: Springer.

[ref051] Taylor, A. L. and Sasser, J. N. 1978. Biology, identification and control of root-knot nematodes (*Meloidogyne* species). Raleigh: North Carolina State University.

[ref052] Tavares-Silva, C. A. , Dias-Arieira, C. R. , Puerari, H. H. , Silva, E. J. D. and Izidoro, A. Jr 2017. Crambe–soybean succession on the management of Pratylenchus *brachyurus* and *Meloidogyne javanica* . Summa Phytopathologica 43:316–320.

[ref053] Wang, D. , Rosen, C. , Kinkel, L. , Cao, A. , Tharayil, N. and Gerik, J. 2009. Production of methyl sulfde and dimethyl disulfde from soil-incorporated plant materials and implications for controlling soilborne pathogens. Plant Soil 324:185–197.

[ref054] Xie, G. , Cui, H. , Dong, Y. , Wang, X. , Li, X. , Deng, R. , Wang, Y. and Xie, Y. 2017. Crop rotation and intercropping with marigold are effective for root-knot nematode (*Meloidogyne* sp.) control in angelica (*Angelica sinensis*) cultivation. Canadian Journal of Plant Science 97:26–31.

